# A mathematical model to predict the change in direction of the common limb in Z plasty

**DOI:** 10.4103/0970-0358.53015

**Published:** 2009

**Authors:** Sunderraj Ellur, Norman L. Guido

**Affiliations:** Department of Plastic, Reconstructive Surgery and Burns, St John's Medical College, Bangalore - 560 034, India

**Keywords:** Z Plasty, Mathematical model, Scar revision

## Abstract

The Z plasty is a common procedure used in scar revision. It is used to break the line of a scar. It can also be used to change the direction of a scar. This article presents a mathematical model to help select an appropriate angled Z to enable the planned change in the direction of the final scar.

## INTRODUCTION

The Z plasty is a fundamental technique in plastic surgery. Over the ages, various modifications of this technique have appeared in literature which concern the extent of elongation of the linear scar,[1] the planimetric properties,[2] and size of the Z in relation to the available laxity.[3] This article attempts to more profoundly evaluate the properties of Z plasty in respect to the change in direction of the common limb in a Z plasty. It is well known that in a classic or 60° Z plasty the common limb rotates by 90°. Using the principles of trigonometry, a study of the angle of rotation of the common limb in other (non-classical) Z plasties is presented.

## MATERIAL AND METHODS

**Figure 1:**
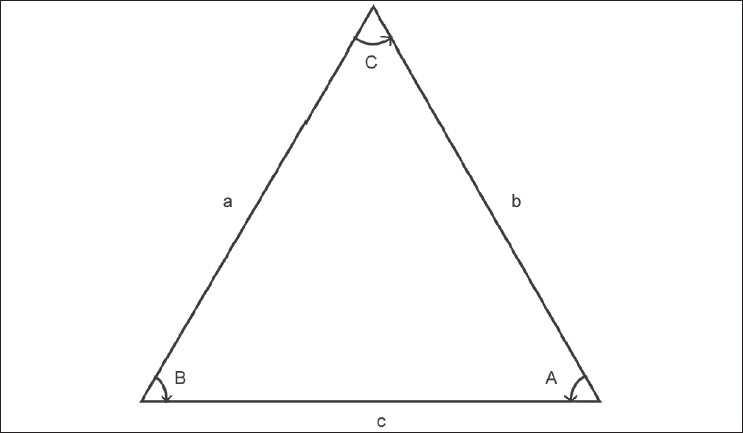
Example of a triangle with three sides a, b, and c and three angles A, B, and C

A triangle has three sides and three angles. Given the values of two sides and the enclosed angle, the values of the other angles can be found using trigonometry, as illustrated. Consider a triangle having three sides a, b, and c and angles A, B, C as shown in Figure 1. If sides a and b and angle C are known, then side c can be found by using the following formula:

$$c^{2}=a^{2}+b^{2}-2\mathrm{ab}[\cos C]\;(1)$$

Furthermore, if the three sides a, b, and c are known, then angle A can be found by using the following formula:

$$\mathrm{cosA}=(b^{2}+c^{2}-a^{2})/2\mathrm{bc}\;(2)$$

**Figure 2:**
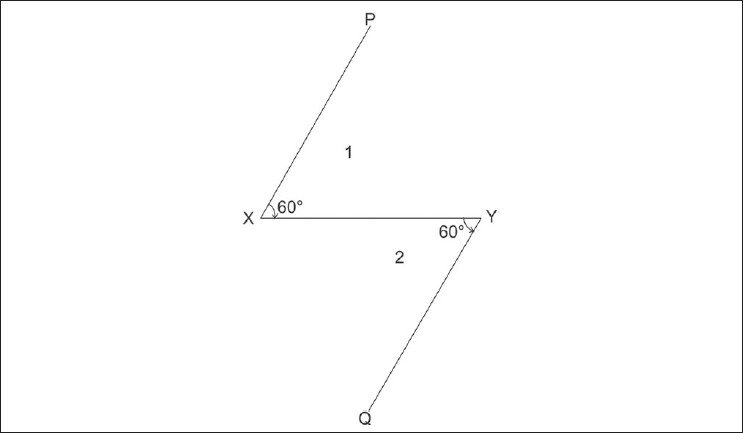
XY represents the scar whose direction is planned to be changed by a classical 60° Z plasty involving Flaps 1 and 2

**Figure 3:**
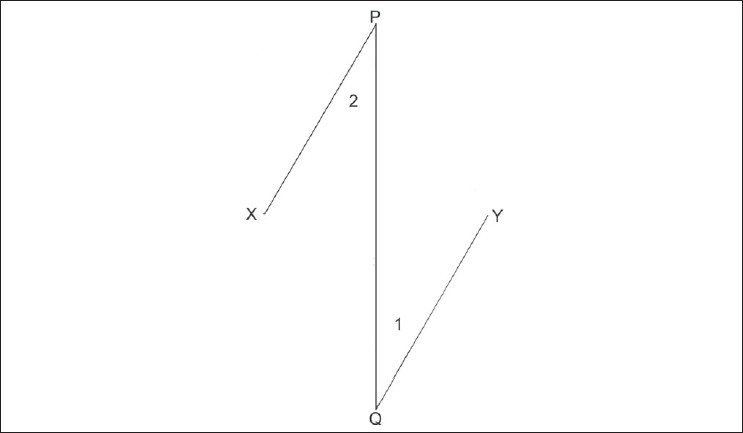
Position of Flaps 1 and 2 after Z plasty transposition

**Figure 4:**
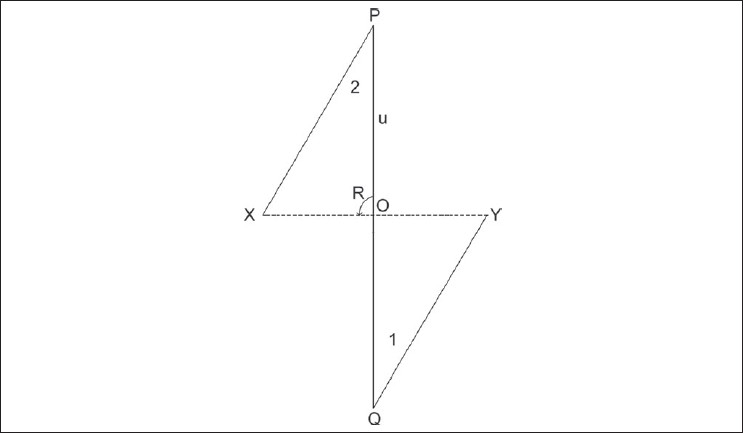
The scar line XY represents the common limb of the Z plasty before flap transposition and line PQ represents the position of the common limb after Flaps 1 and 2 are transposed

Now, consider a classical 60° Z plasty. Although we all know that the common limb moves by 90°, let's confirm it mathematically using the above formula. In Figure 2, XY represents the scar whose direction is planned to be changed by a classic 60° Z plasty. Lin es PX and YQ are drawn so that angle PXY and angle XYQ are 60°, resulting in Flaps 1 and 2. Figure 3 shows the position of Flaps 1 and 2 after Z plasty transposition. To enable the application of the principles of trigonometry to our problem, the dashed line XY, representing the original scar line, is made as shown in Figure 4. Thus, the scar line XY represents the common limb of the Z plasty before flap transposition and line PQ represents the position of the common limb after Flaps 1 and 2 are transposed. The point where the two common limbs intersect will be called O. The angle XOP represented as R in the diagram measures the degree of rotation of the common limb in a 60° Z plasty. Let the limbs of the described 60° Z plasty be 2 cm to facilitate easier calculation using trigonometry (the results achieved will be similar irrespective of the length of the limbs chosen, since the angle of the rotation is independent of the length of the limb). Then, consider the triangle XOP in Figure 4. Line XP will be 2 cm, XO will be 1 cm, and PO will be U cm. The value of R can be derived using trigonometry by calculation in two parts. The first part of the calculation involves deriving the value of U by using the formula 1 above. In the second part of the calculation, having known the value of U, the value of R can be derived using formula 2 above. The functional connection between the angle in a Z plasty (C) and the common limb angle of rotation (R) can also be calculated using the simple formula R = arcos[(0.5-cosC)/√(1.25-cosC)].

### Part 1:

Formula is

c2=a2+b2-2ab[cosC]

Hence,

U^2^ = 2^2^ + 1^2^ (2 × 2 × 1) cos 60

U^2^ = 4 + 1 - 4 cos 60

U^2^ = 5 - 4 cos 60   [cos 60 = 0.5]

U^2^ = 5 - 4 × 0.5

U^2^ = 5 - 2

U^2^ = 3

U = √3

U = 1.73

### Part 2:

Formula is

cosA=(b2+c2-a2)/2bc

Hence,

cos R = (1^2^ + (1.73^2^ - 2^2^)/2bc

cos R = (1 + 3 - 4)/3.46

cos R = 0/3.46

cos R = 0

R = arcos 0

R = 90° ................................ [cos Inverse of 0 = 90°]

Thus, it is confirmed mathematically that in a 60° Z plasty the common limb rotates by 90°.

The same methodology was followed to identify the angle of rotation of the common limb in various other angled Z plasties. The values derived were then tabulated in Table 1.

**Table 1:** The degree of rotation for various angled Z plasties

*Angle of z plasty*	*Angle of rotation of common limb*
30	126.2
35	119.1
40	112.5
45	106.3
50	101
55	95.1
60	90
65	85.1
70	80.5
75	76
80	71.7
85	67.5
90	63.5

### DISCUSSION

**Chart 1:**
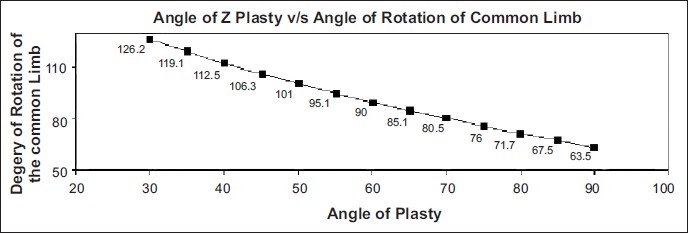
The curve of Alexander Limberg

The Z plasty is a procedure that involves the transposition of two interdigitating triangular flaps. In 1856, Denonvilliers described the first Z plasty.[4] However, the geometrical principles were beautifully described by Alexander Limberg in 1929 [Chart 1].[5] The Z plasty is a technique often used in scar revision. Among the reasons why it is useful in scar revision is its ability to make the revised scar lie in or parallel to a crease line or relaxed skin tension line. This is due to the rotation of the revised scar by a particular degree depending on the angle of the Z used. It is known that the limbs of the Z can extend at various angles from 30° to 90°.[6] In this article, a mathematical study of the degree of rotation for various angled Z plasties has been made and tabulated as shown in Table 1. This table will be useful in choosing the appropriate Z that will be required having measured the degree of rotation expected from the revised scar. For example, consider a scar that is at 75° to the nasolabial fold, then a 75° Z would be the appropriate Z to be used since in this Z the revised scar is expected to mathematically rotate by 75° [Table 1] and lie in the nasolabial fold.

Another important mathematical finding from this study is that there is a well-defined mathematical relationship between the angle of Z and the rotation of the common limb as shown in Table 1. If one plots the various angles of Z on the X axis and the derived rotation angles on the Y axis, one finds that the values will all fall on a regular curved line, which we may name as “The curve of Limberg” in memory of the great genius. This study is a mathematical study; since the skin doesn't behave as a mathematical model as was confirmed by Furnas, *et al.*, one needs to find a method to accurately measure the amount of change in the direction of the common limb in skin.[7]
